# Mental health and financial concerns in medical students: insights from a cross-sectional study in Germany

**DOI:** 10.1186/s12909-025-07723-5

**Published:** 2025-10-02

**Authors:** Anne Jenet, Judith Tillmann, Jan Ehlers, Kerstin Herzog, Julia Nitsche, Klaus Weckbecker, Eva Münster

**Affiliations:** 1 Institute of General Practice and Primary Care, Chair of General Practice I and Interprofessional Care, Witten/Herdecke University, Alfred-Herrhausen-Str. 50, Witten, 58455 Germany; 2 Chair of Didactics and Educational Research in Health Care, Faculty of Health, Witten/Herdecke University, Witten, Germany; 3 Department of Social Work, Hochschule RheinMain, Wiesbaden, Germany

**Keywords:** Financial toxicity, Psyche, Debt, Financial concerns, University, Medicine

## Abstract

**Background:**

Numerous students in Germany are affected by mental health disorders. International studies indicate that financial concerns represent a significant risk factor for the onset of mental health issues among students. Medical students may be particularly vulnerable due to the prolonged and often demanding nature of their studies. However, there is currently no data on this topic in Germany. The aim of this study was to investigate the prevalence of financial concerns among medical students and whether there is an association with their mental health.

**Methods:**

From April to May 2024, an anonymous cross-sectional study was conducted among all medical students at a private university in Germany using an online questionnaire. The data were analysed using descriptive statistics and binary logistic regression.

**Results:**

A total of 663 medical students participated in the study (response rate: 63.4%), of whom 564 participants (54.0%) were included in the analysis. 44.7% of students reported below-average mental health, and an additional 22.7% were severely impaired mental health. 55.7% reported financial concerns, and 23.6% had debts. Students with financial concerns were approximately three times more likely to be mentally impaired (32.8%) compared to students without financial concerns (10.1%). Compared to men, women had almost four times higher odds of experiencing mental impairment. Additionally, students with a moderate to very poor general physical health status had more than three times higher odds of having below-average mental health (aOR = 3.31; 95% CI: 1.60–6.85). Financial concerns also increased the likelihood of having below-average (aOR = 2.04; 95% CI: 1.33–3.14) or impaired mental health (aOR = 6.20; 95% CI: 3.19-12.00).

**Conclusion:**

For the first time in Germany, this study uncovered widespread financial concerns, debt, and significant correlations between financial concerns and poor mental health among medical students at a private university. There is an urgent need for further research to better understand the causes and consequences of this issue and to develop targeted countermeasures and support services at both the political and university levels. Early counselling and therapy options addressing both mental health challenges and financial concerns are crucial for mitigating their impact on academic performance and ensuring the well-being of future medical professionals.

**Supplementary Information:**

The online version contains supplementary material available at 10.1186/s12909-025-07723-5.

## Background

Students represent a population group in a crucial stage of development that will have a significant impact on their future [1]. The university years are considered a peak period for the onset of mental health disorders, such as depression [2]. A nationwide U.S. survey found that nearly half of all final-year students had a mental health disorder affecting their daily functioning [3]. A Systematic Review among U.S. and Canadian Medical Students suggest a high prevalence of depression and anxiety among medical students, with levels of overall psychological distress consistently higher than in the general population and age-matched peers by the later years of training [4]. In Germany, the “best3” study involving 188,000 students from 250 universities showed a significant increase in the number of students reporting mental health issues, from 45 to 65% between 2011 and 2021 [5].

Mental health disorders can negatively affect physical, mental, and social functioning, thereby reducing overall quality of life [6]. U.S. studies report that such issues can reduce university attendance and decrease the likelihood of successfully completing one’s studies [3], as well as negatively affect overall educational outcomes [7]. A 2021 cross-sectional study in Germany, which examined 187,935 students from 250 universities, found that nearly 24% of students were health-impaired, with almost 16% reporting that at least one health issue adversely affected their studies [8]. Among these, mental health disorders were particularly widespread, with 65% of these students reporting such conditions [8].

Research suggests that financial stress and difficulties (referred to hereafter as “financial concerns”) is associated with poorer mental and physical health among students. In a quantitative study from the UK, financial concerns were a significant predictor of poorer health, including physical functioning, social functioning, mental health, energy/vitality, pain, and overall health perception [9]. According to a systematic review with meta-analysis, financial concerns among students also increase the risk of developing depression and suicidal events [10]. A UK study found that financial difficulties significantly impacted not only depression but also relationship problems and anxiety disorders [11]. British students with high financial concerns reported feeling “more tense, anxious, or nervous” and “criticized by others,” and had more “difficulty falling or staying asleep”, compared to those with lower financial concerns [12]. A longitudinal analysis by Richardson et al. [13] showed that financial difficulties in the first year of university increased the risk of psychosis over time, both in terms of symptoms and distress.

Medical students in Germany face a particularly demanding and lengthy educational path, requiring 12–13 years of regular study time, plus an additional five to six years of specialization training, with high entry requirements. Although tuition fees at public universities in Germany have been abolished, students still face semester fees, and private universities impose substantial tuition costs. A systematic review indicates that medical students exhibit high levels of financial concern, often associated with debts [14]. These debts also led to lower academic performance on average and poorer mental health among medical students [14].

There is a lack of research on the relationship between financial concerns and mental health among students in Germany. Although mental health and financial concerns have been researched among students in Germany [8], the relationship between these factors has not been explored. Moreover, the specific situation of medical students has to be considered. Given the current gap in knowledge in Germany on this topic, it was crucial to conduct a primary study to investigate this issue for the first time. The “Financial Concerns Health Student Study” (finCoHST study) aims to examine the prevalence of mental health impairment and financial concerns among medical students at a private university in Germany and to explore whether there is a relationship between these factors.

Our study focuses on mental health, not mental disorders, using the MHI-5, a short, valid, and reliable international tool to assess it [15]; mental health is a state of well-being in which individuals are emotionally stable, think clearly, interact effectively with others, and are resilient to everyday stress. It enables people to realize their abilities, work productively, and contribute to their community. Mental disorders are diagnosable conditions like depression, anxiety, or eating disorders. These definitions are based on the World Health Organization’s (WHO) [16, 17]. 

## Methods

### Study design

The finCoHST study was designed as an anonymous cross-sectional survey conducted from early April to mid-May 2024 among medical students at a private university in Germany. Primary data were collected online using LimeSurvey. The survey was integrated as a module into the semi-annual student survey for the summer semester of 2024. All 1,045 medical students enrolled at the time of data collection received a link to the questionnaire. Three reminders were sent at one-week, two-week, and three-week intervals, with the survey closing after five weeks.

Ethical approval was obtained from the university of the study collaborators for the finCoHST study (No. S-282/2023).

### Study population

The inclusion criteria were current enrollment as a medical student at the studied private university at the time of data collection (*n* = 1,045).

### Survey instrument

An information text with a privacy statement and a questionnaire consisting of 42 questions were developed for the finCoHST study. The questionnaire was developed by the interdisciplinary study team based on literature research. Individual questions as well as groups of questions from previously conducted surveys by other scientific and research groups were incorporated into the questionnaire. The questionnaire was administered in german. The questionnaire covered five main topics: sociodemographic characteristics, study-related aspects, financial situation, general physical health, mental health. For the data analyses presented here, the specific questions are available as supplementary material (appendix 1).

The validated Mental Health Inventory (MHI-5) [18], German version [19], was used as an indicator to assess mental health. The five questions listed in appendix 1 were answered using predetermined response options. The question block on financial concerns listed in appendix 1 was adopted after consulting Donna Jessop, the author of the following publications: “Financial concern predicts deteriorations in mental and physical health among university students, 2019” [20] and “The impact of financial circumstances on student health, 2005” [9]. The questionnaire was translated from English into German, and the accuracy of the translation was verified by independent third parties through back-translation into English.

Completing the entire questionnaire took no longer than ten minutes. A pretest phase was conducted with ten test participants. Minor spelling and typographical errors were corrected, and the options for specifying the current semester were adjusted. Feedback indicated that the questionnaire was easy to understand and user-friendly.

### Statistical analysis

The data were transferred to IBM SPSS Statistics, version 29, for plausibility checks, preparation, and exploratory analysis using univariate, bivariate, and multivariate analysis. A significance level with a probability of error of α = 0.05 was set. All participants who indicated they were not studying medicine (*n* = 28) were excluded from the dataset. In the descriptive analyses, missing values were treated as a separate category “Missing”. Continuous variables were analyzed using t-tests (for two groups) or ANOVA (for more than two groups) after confirming normal distribution with the Kolmogorov-Smirnov test. Chi-square tests were used for examining relationships between categorical variables.

The “Mental Health” variable was calculated based on the Mental Health Inventory (MHI-5): The sum of the five items yielded raw scores between 5 and 25. The score was then calculated using the formula ((raw score − 5) x 5), yielding values between 0 and 100 [21]. A three-part analysis followed based on the cut-off values derived from a study published by the Robert Koch Institute (RKI) [15]: MHI Score: 75–100 was classified as “average or better” mental health. MHI Score: 51–74 was classified as “below average” mental health. MHI Score ≤ 50 was classified as “impaired” mental health. Missing responses for any of the five items in the MHI-5 were recoded as “not exposed” (*n* = 10). Participants who answered fewer than four items were excluded (*n* = 71).

A mean score was calculated from the six items of “financial concerns”, which could range from 1 to 7. A higher score indicated greater financial concern [20]. In addition to the Mean Score, and independent of the original publication [9, 20], a Risk Score for financial concerns was calculated by dichotomizing the variable. Financial concerns were defined as present if at least one of the six items was answered with “strongly agree”, “agree”, or “somewhat agree”. If there was a missing value in any of the six items, it was recoded as “strongly disagree” (*n* = 25), and participants who answered fewer than five items were excluded (*n* = 12).

Binary logistic regression analyses were performed using mental health as the dependent variable. The following sociodemographic, socioeconomic, and health-related variables were considered as independent variables: sex by birth certificate (male, female), age (in years), migration background, number of semesters, highest completed vocational qualification (university degree, vocational training, none), financial debt (yes, no), and general physical health (“very good, good” vs. “moderate to very poor”). A migration background was defined, according to the Federal Statistical Office in Germany (“Destatis”), as having been born in a country other than Germany or having at least one parent born outside of Germany [22]. Adjusted odds ratios (aOR) with 95% confidence intervals were calculated. Missing values in the categorical independent variables in the logistic regression model were assigned to the reference category with the largest n as long as they were below 5% (sex by birth certificate: *n* = 2, migration background: *n* = 10, completed studies or vocational training: *n* = 2, financial debt: *n* = 27, general physical health: *n* = 3). For missing continuous variables, mean imputation was used (age: *n* = 4, semester: *n* = 1, financial concerns: *n* = 12). A logistic regression model was fitted using the enter method (i.e., all predictors were included in a single step). Two regression models were constructed, categorizing the dependent variable as follows: (a) Below-average mental health vs. healthy (average or better) and (b) Impaired mental health vs. healthy. This was done to show a gradient in risk strength that makes clinical relevance understandable.

## Results

### Characteristics of the study population

A total of 663 medical students participated. Of these, *n* = 542 fully completed the questionnaire, while *n* = 121 submitted partial responses (response rate: 63.4%). The sociodemographic and -economic characteristics of the study sample are presented in Table 1. Data from 564 medical students, aged between 18 and 53 years (M = 24.7; SD = 3.4), were analyzed. (*n* = 28 indicated that they were not studying at the private University of this study and *n* = 71 no MHI score could be obtained). Approximately one-third of the participants were male and two-thirds were female. The students were in semesters 1 to 13 (M = 5.4; SD = 3.2). Nearly one in five participants had a migration background. 23.6% of the participants reported having debts.

### Mental health

Table 1 also presents the distribution of these sociodemographic and -economic variables stratified by mental health (MHI-5). Overall, approximately one-third of the sample reported good mental health, while nearly 45% had below-average mental health, and one in five participants reported impaired mental health. Women were significantly more likely than men to report impaired mental health.

**Table 1:** Sociodemographic and socioeconomic characteristics of the study sample stratified by mental health (collected by MHI-5), finCoHST study (n=564)

	**Total**		**Mental health **						***p*** **-value**
			Healthy (average or better)		Below average		Impaired		
	*n*	%^1^	*N*	%^2^	*n*	%^2^	*n*	%^2^	
Total	564	100	184	32.6	252	44.7	128	22.7	
Sex (birth certificate)									<0.001
Female	374	66.3	93	24.9	182	48.7	99	26.5	
Male	188	33.3	91	48.4	69	36.7	28	14.9	
Missing	2	0.4	0	0.0	1	50.0	1	50.0	
Age (years)									0.023
Mean±SD (Median)*	24.7±3.4 (24)		24.5±3.8 (24)		24.5±3.1 (24)		25.4±3.4 (26)		
Missing	4	0.7	1	25.0	0	0.0	3	75.0	
Migration background									0.233
No	453	80.3	150	33.1	209	46.1	94	20.8	
Yes	101	17.9	30	29.7	40	39.6	31	30.7	
Missing	10	1.8	4	40.0	3	30.0	3	30.0	
Number of semesters									0.522
Mean±SD (Median)	5.4±3.2 (5.0)		5.3±3.1(5.0)		5.5±3.2 (5.0)		5.2±3.2 (5.0)		
Minimum; Maximum	1.0; 13.0		1.0; 13.0		1.0; 12.0		1.0; 12.0		
Missing	1	0.2	0	0.0	1	100.0	0	0.0	
Highest completed professional qualification									0.261
(University) studies	52	9.2	18	34.6	21	40.4	13	25.0	
Vocational training	257	45.6	74	28.8	113	44.0	70	27.3	
None	253	44.9	92	36.4	117	46.3	44	17.4	
Missing	2	0.4	0	0.0	1	50.0	1	50.0	
Financial debts									0.002
Yes	133	23.6	31	23.3	56	42.1	46	34.6	
No	404	71.6	146	36.1	181	44.8	77	19.1	
Missing	27	4.8	7	25.9	15	55.6	5	18.5	
Mean-Score for financial concerns									<0.001
Mean±SD (Median)	2.9±1.3 (2.8)		2.3±1.3 (2.0)		2.9±1.3 (2.8)		3.9± 1.4 (3.8)		
Minimum; Maximum	1.0; 6.8		1.0; 5.5		1.0; 6.3		1.0; 6.8		
Missing	12	2.1	5	41.7	6	50.0	1	8.3	
Risk-Score for financial concerns									<0.001
No risk	238	42.2	109	45.8	105	44.1	24	10.1	
Under risk	314	55.7	70	22.3	141	44.9	103	32.8	
Missing	12	2.1	5	41.7	6	50.0	1	8.3	
General physical health									<0.001
Very good, good	447	79.3	171	38.2	205	45.7	71	15.9	
Moderate to very poor	114	20.2	11	9.7	46	40.4	57	50.0	
Missing	3	0.5	2	66.7	1	33.3	0	0.0	

^1^column percentage

^2^row percentages

*minimum and maximum not provided due to data protection reasons

On average, students with impaired mental health were nearly one year older than those in the other groups, and that difference was statistically significant. A significant correlation was found between general physical health and mental health: Half of participants with average to very poor general physical health reported impaired mental health, whereas only about 16% of those with very good or good general physical health experienced mental health impairments.

A significant correlation was also observed between financial situation, including being in debt, and mental health: the mean score showed that increasing financial concerns were associated with a tendency toward poorer mental health. This finding was reinforced by the financial concerns risk variable: 30.8% of the students with risk for financial concerns showed impaired mental health while those without risk for financial concerns did it in 10.1%. of cases.

Table 2 presents the adjusted risk estimates for the associations between influencing factors and mental health.

**Table 2:** Mental health (collected by MHI-5) and sociodemographic/-economic factors: Adjusted Odds Ratios (aOR) with 95% confidence intervals; finCoHST (*n*=564)

	**Model I: Mental health: below average (1) versus healthy (0)**		**Model II: Mental health: impaired (1) versus healthy (0)**	
	aOR	95% CI	aOR	95% CI
Sex (birth certificate)				
Female	Ref		Ref	
Male	0.36	0.24-0.56	0.25	0.13-0.48
Age (years)	0.97	0.91-1.05	1.01	0.92-1.11
Migration background				
No	Ref		Ref	
Yes	0.96	0.55-1.70	1.40	0.68-2.90
Number of semesters	1.01	0.96-1.08	1.03	0.94-1.12
Highest completed professional qualification				
None	Ref.		Ref.	
(University) studies	0.90	0.37-2.02	1.13	0.38-3.35
Vocational training	1.20	0.74-1.93	1.36	0.68-2.70
Financial debts				
No	Ref.		Ref.	
Yes	1.20	0.68-2.10	1.38	0.68-2.81
Risk-Score for financial concerns				
No risk	Ref.		Ref.	
Under risk	2.04	1.33-3.14	6.20	3.19-12.00
General physical health				
Very good, good	Ref.		Ref.	
Moderate to very poor	3.31	1.60-6.85	12.45	5.51-28.12

*Ref* =Reference category; Missings were assigned to the reference category

Several predictors for below-average or impaired mental health were identified. Significant factors included sex, the presence of financial concerns (risk score), and general physical health status. Women had nearly four times the odds of experiencing impaired mental health compared to men. Financial concerns, as measured by the risk score, were associated with twice the odds of below-average mental health (aOR = 2.04; 95% CI: 1.33–3.14). The odds for impaired mental health were more than six times higher for students with financial concerns (aOR = 6.20; 95% CI: 3.19–12.00). Students with an average to very poor general physical health status had more than three times the odds of below-average mental health (aOR = 3.31; 95% CI: 1.60–6.85) and twelve times the odds of impaired mental health (aOR = 12.45; 95% CI: 5.51–28.12), compared to those with very good or good physical health.

No significant association with mental health impairment was found for the predictors age, migration background, semester, completed professional degree, and financial debt in the model.

## Discussion

### Summary

This study is the first in Germany to identify widespread financial concerns (55.7%) and financial debt (23.6%) among medical students, as well as significant associations between financial concerns and poor mental health at a private university in western Germany. In addition to women and students with a moderate to very poor general physical health status, those experiencing financial concerns had significantly higher odds of having below-average (aOR = 2.04; 95% CI: 1.33–3.14) or impaired mental health (aOR = 6.20; 95% CI: 3.19-12.00).

### Mental health of students

As described in the background, university years are a critical period for mental health, where factors such as academic pressure, financial burdens, and social challenges could increase the risk. The portion of students reporting impaired mental health in our study (22.7%) is comparable to international findings from the World Health Organization (WHO) World Mental Health Surveys conducted in 21 countries, which reported that 20.3% of 1,572 college students met the criteria for a DSM-IV disorder [23]. In the general German population, the 12-month prevalence of mental disorders was 27.7% in a representative national cohort aged 18 to 79 years, further highlighting the similarity to our findings [24]. Adding the number of students with below-average mental health in our study, 67.4% were suffering, similar to the results of the best3-study in Germany for 2021, which reported 65% of university students with mental health issues [5]. To improve the frequent mental health issues among students, particularly in the medical field, targeted measures like preventive approaches should be developed. Easily accessible information and counselling services could help identify and address mental health concerns in a timely manner. For medical students, who are particularly affected by the intense training and frequent emotional burdens, mental health should be more strongly integrated into the curriculum to encourage open discussion and facilitate access to professional support. Additionally, structures should be created to reduce overall stress for students. It is crucial to destigmatize mental health issues and establish a culture of openness and support. Closer collaboration with psychological and psychiatric professionals could help reduce students’ reluctance to seek help when needed.

### Prevalence of financial concerns among medical students

The prevalence of financial concerns (55.7%) and financial debt (23.6%) among medical students in this study are difficult to compare due to the lack of similar studies in Germany and differing tuition fee systems internationally. In Germany, most medical universities are tax-funded and, therefore, tuition-free, with the exception of private universities like the one in this study [25]; there are 109 public universities in Germany, 39 of which offer programs in human medicine, while 9 private universities also offer medical studies.

In a study of 7,203 students from various fields conducted at five German universities during the fourth wave of the COVID-19 pandemic, 13.9% reported insufficient financial resources to cover their monthly expenses [26]. High levels of financial concerns specifically among medical students can only be compared with international studies, where similar findings have been reported. A systematic review, predominantly based on studies from the USA, as well as some from Canada, New Zealand, Scotland, and Australia, indicated high levels of financial stress among medical students, which were correlated with debt. Finally, debt was also associated with poorer academic performance and negatively impacted mental well-being [14]. International studies in the USA, UK and Australia also report financial difficulties as a common issue among medical students: Financial problems are a frequent reason for students to suspend or consider suspending their studies [27]. In a UK study, 37.7%, of respondents stated that worrying about money affected their studies [28]. In an US cross-sectional study, 16% of medical students reported education-related debt [29], while another study found that 71% were concerned about the financial burden associated with medical school [30]. The German student units (deutsche Studierendenwerke) point to the social polarization of student financing, as more than a third of students, for example, have less than 800 euros per month at their disposal [31]. At the same time, rent expenses and living costs, e.g. for food and healthcare, are rising [8]. BAföG funding rates, the most important instrument of state student funding, have been steadily declining since 2016 [8]. Increases in BAföG rates and parental allowances are being demanded [31].

In 2022, the average monthly BAföG amount per student was 611 euros, according to the Federal Statistical Office in Germany. The exact amount depends on factors such as housing costs, as well as the income and assets of the student and their family members [32].

BAföG is a key source of financial support for students at both private and public universities. This is especially relevant for medical students, whose long and demanding studies leave little time for parttime jobs, especially during the low paid Practical Year. In addition, they also have to cover living and housing expenses.

Financial concerns and debt may be more prevalent in this study due to the private university setting with tuition fees and the extended duration of medical training. Further research on this topic is needed in Germany to gain a clearer picture.

### Association between financial concerns and mental health

Although there are no studies examining the relationship between financial concerns and mental health among German medical students, some international studies have shed light on this association among students in general: A British study of 454 undergraduate students found that greater financial difficulties predicted poorer mental health and higher levels of anxiety, depression, stress and alcohol dependence [33]. In a systematic review and meta-analysis by Sheldon et al. [10], undergraduate students with financial difficulties had an increased risk of depression and suicide-related outcomes (pooled OR 1.83; 95% CI: 0.71–4.69). A German cross-sectional study, conducted at five German universities (*n* = 7,203), found that a worsened financial situation at the time of the survey, compared to prior to the COVID-19-pandemic, was associated with higher levels of anxiety and depressive symptoms [26].

Increasing evidence suggests that the amount of stress about debt, rather than debt itself, may be the primary factor affecting mental health [34, 35]. Possible reasons could be that students anticipate future debt from tuition fees or that current concerns about living costs outweigh existing loan amounts. In a longitudinal study among UK students, greater subjective stress about debt worsened mental health over time, including anxiety and depression [12]. Other British studies among students found that increased financial concerns were significantly associated with poorer mental and physical health [9, 20]. Another British study with similar results also highlights the negative impact of mental health problems on academic performance [11]. The existing literature in this field is limited and warrants further investigation among students [33]. The consequences of financial concerns and mental health issues during this important phase of life, as well as measures to counteract them, should urgently be investigated.

### Sex, physical and mental health

Our findings, which show that women had higher odds of reporting mental health issues, align with the broader literature indicating a greater proportion of females experiencing common mental health difficulties [33, 36], as well as specific mental illnesses such as anxiety disorders [37, 38]. This is likely not attributable to sex itself, but rather to gender-specific differences in self-disclosure, help-seeking behaviour, and the utilization of health services [10, 39]. Female students may therefore be more inclined to discuss their problems and seek treatment. These findings underscore the importance of recent efforts to tailor services to those actively seeking help, while also creating proactive strategies to engage students who are less likely to seek support [40]. It should be noted that our study only takes into account sex assigned at birth. The correlation between physical and mental health has been extensively researched in different contexts [41–43], and our study supports this as well. Therefore, it will not be explored further.

### Strengths and limitations

A key strength of this study is its sample size (*n* = 564 medical students), as well as the high response rate. Additionally, the anonymity of the survey contributes to unbiased responses. The study benefits from the use of standardised measures for both financial and mental health variables. However, the potential for selection bias must be considered: On the one hand, students experiencing psychological distress and financial concerns may have been more likely to participate in order to express their situation. On the other hand, it is also conceivable that students with severe psychological distress and significant financial concerns refrained from participating due to a lack of energy, endurance, or because of feelings of shame. As a cross-sectional study, causality cannot be established. It remains unclear whether financial concerns are the cause of psychological distress or whether psychological distress, in turn, exacerbates financial concerns. Furthermore, our approach to missing data may have caused a bias in the risk estimators toward zero effect. A survey with a larger study population and complete data is desirable. Future research should employ longitudinal studies to examine causal relationships. Furthermore, the generalizability of the findings must be discussed. This study included only medical students from a private university. To assess generalizability, further research is needed, including both private and public universities and different subjects of study. Future studies could also consider the different admission criteria between public and private universities. Public ones typically select based on high school grades, while private institutions often use university specific procedures. Possible differences between private and public universities regarding financial concerns and mental health should be considered. At private universities, students may face higher financial burdens due to tuition fees, in addition to other debts such as BAföG or student loans. While it is possible that private university students come from higher income families, this varies depending on the university’s financing model for example the private University in this study offers income dependent repayment through a reversed generational contract. Without such models, reliance on family financial support may be greater. Private universities may have smaller cohorts and may offer more personalized support, which could benefit mental health. At the same time, tuition fees might increase the pressure to continue studying despite personal difficulties, as dropping out could feel like a financial loss.

It would be interesting to also collect data on social background as an aspect affecting students’ financial resources, as well as household structure. Additionally, it would be valuable to examine whether students are employed alongside their studies and how such employment influences their financial situation and mental health. It would also be valuable to examine whether students are first-generation students, meaning they are the first in their family to attend university.

## Conclusions

There is an urgent need for further research on financial concerns and mental health among students in Germany to better understand their causes and consequences. Based on these findings, targeted interventions and support services should be developed. Preventive approaches, early access to information and professional financial and psychological support at universities, stronger integration of mental health into the curriculum, as well as efforts to destigmatize mental health issues and reduce overall stress, could be beneficial. In concrete terms, free financial counselling services at universities, offering regular consultation hours to support students with individual financial planning, BAföG, student loans, and related questions, should be established. Students form a crucial pillar of tomorrow’s society and deserve greater attention, particularly those in medical education.

## Supplementary Information

Supplementary Material 1.

## Data Availability

The datasets used and analyzed during the current study are available from the corresponding author on reasonable request.

## Abbreviations

aOR: Adjusted Odds Ratio
CI: Confidence interval
finCoHST: Financial Concerns Health Student Study
MHI-5: Mental Health Inventory
M: Mean
SD: Standard deviation
WHO: World Health Organization

## References

[1] Ge Y, Xin S, Luan D, Zou Z, Liu M, Bai X, Gao Q. Association of physical activity, sedentary time, and sleep duration on the health-related quality of life of college students in Northeast China. Health Qual Life Outcomes. 2019;17:124. 10.1186/s12955-019-1194-x. 31311564 10.1186/s12955-019-1194-x PMC6636029

[2] Ibrahim AK, Kelly SJ, Adams CE, Glazebrook C. A systematic review of studies of depression prevalence in university students. J Psychiatr Res. 2013;47:391–400. 10.1016/j.jpsychires.2012.11.015. 23260171 10.1016/j.jpsychires.2012.11.015

[3] Blanco C, Okuda M, Wright C, Hasin DS, Grant BF, Liu S-M, Olfson M. Mental health of college students and their non-college-attending peers: results from the National epidemiologic study on alcohol and related conditions. Arch Gen Psychiatry. 2008;65:1429–37. 10.1001/archpsyc.65.12.1429. 19047530 10.1001/archpsyc.65.12.1429 PMC2734947

[4] Dyrbye LN, Thomas MR, Shanafelt TD. Systematic review of depression, anxiety, And other indicators of psychological distress among U.S. And Canadian medical students. Acad Med. 2006;81(4)354–373. 10.1097/00001888-200604000-00009 16565188

[5] Deutsches Zentrum für Hochschul- und Wissenschaftsforschung GmbH (DZHW), Deutsches Studentenwerk (DSW). Die Studierendenbefragung in Deutschland: best3: Studieren mit einer gesundheitlichen Beeinträchtigung. 2023. https://www.studierendenwerke.de/fileadmin/user_upload/Publikationen/beeintraechtigt_studieren_2021.pdf. Accessed 28 Jan 2025.

[6] Herbert C. Enhancing mental health, Well-Being and active lifestyles of university students by means of physical activity and exercise research programs. Front Public Health. 2022;10:849093. 10.3389/fpubh.2022.849093. 35548074 10.3389/fpubh.2022.849093 PMC9082407

[7] King KM, Meehan BT, Trim RS, Chassin L. Marker or mediator? The effects of adolescent substance use on young adult educational attainment. Addiction (Abingdon England). 2006;101:1730–40. 10.1111/j.1360-0443.2006.01507.x. 17156172 10.1111/j.1360-0443.2006.01507.x PMC2238681

[8] Deutsches Zentrum für Hochschul- und Wissenschaftsforschung GmbH (DZHW). Die Studierendenbefragung in Deutschland: 22. Sozialerhebung: Die wirtschaftliche und soziale Lage der Studierenden in Deutschland 2021. 2023. https://www.bmbf.de/SharedDocs/Publikationen/DE/4/31790_22_Sozialerhebung_2021.pdf?__blob=publicationFile&v=4. Accessed 28 Jan 2025.

[9] Jessop DC, Herberts C, Solomon L. The impact of financial circumstances on student health. Br J Health Psychol. 2005;10:421–39. 10.1348/135910705X25480. 16238857 10.1348/135910705X25480

[10] Sheldon E, Simmonds-Buckley M, Bone C, Mascarenhas T, Chan N, Wincott M, et al. Prevalence and risk factors for mental health problems in university undergraduate students: A systematic review with meta-analysis. J Affect Disord. 2021;287:282–92. 10.1016/j.jad.2021.03.054. 33812241 10.1016/j.jad.2021.03.054

[11] Andrews B, Wilding JM. The relation of depression and anxiety to life-stress and achievement in students. Br J Psychol. 2004;95:509–21. 10.1348/0007126042369802. 15527535 10.1348/0007126042369802

[12] Cooke R, Barkham M, Audin K, Bradley M, Davy J. Student debt and its relation to student mental health. J Furth High Educ. 2004;28:53–66. 10.1080/0309877032000161814.

[13] Richardson T, Yeebo M, Jansen M, Elliott P, Roberts R. Financial difficulties and psychosis risk in British undergraduate students: a longitudinal analysis. JPMH. 2018;17:61–8. 10.1108/JPMH-12-2016-0056.

[14] Pisaniello MS, Asahina AT, Bacchi S, Wagner M, Perry SW, Wong M-L, Licinio J. Effect of medical student debt on mental health, academic performance and specialty choice: a systematic review. BMJ Open. 2019;9:e029980. 10.1136/bmjopen-2019-029980 PMC6609129 31270123

[15] RKI. Daten und Fakten: Ergebnisse der Studie Gesundheit in Deutschland aktuell 2010. 2010.

[16] Mental disorders. 10.06.2025. https://www.who.int/news-room/fact-sheets/detail/mental-disorders. Accessed 10 Jun 2025.

[17] Mental health. 07.06.2025. https://www.who.int/data/gho/data/themes/theme-details/GHO/mental-health. Accessed 10 Jun 2025.

[18] Berwick DM, Murphy JM, Goldman PA, Ware JE, Barsky AJ, Weinstein MC. Performance of a five-item mental health screening test. Med Care. 1991;29:169–76. 10.1097/00005650-199102000-00008. 1994148 10.1097/00005650-199102000-00008

[19] Robert Koch-Institut. Daten und Fakten: Ergebnisse der Studie Gesundheit in Deutschland aktuell 2010: Beiträge zur Gesundheitsberichterstattung des Bundes. Berlin; 2012.

[20] Jessop DC, Reid M, Solomon L. Financial concern predicts deteriorations in mental and physical health among university students. Psychol Health. 2020;35:196–209. 10.1080/08870446.2019.1626393. 31181966 10.1080/08870446.2019.1626393

[21] Rumpf HJ, Meyer C, Hapke U, John U. Screening for mental health: validity of the MHI-5 using DSM-IV axis I psychiatric disorders as gold standard. Psychiatry Res. 2001;105:243–53. 10.1016/S0165-1781(01)00329-8. 11814543 10.1016/s0165-1781(01)00329-8

[22] Statistisches Bundesamt. Migrationshintergrund: Definition. 26.02.2020. https://www.destatis.de/DE/Themen/Gesellschaft-Umwelt/Bevoelkerung/Migration-Integration/Glossar/migrationshintergrund.html. Accessed 10 Jun 2025.

[23] Auerbach RP, Alonso J, Axinn WG, Cuijpers P, Ebert DD, Green JG, et al. Mental disorders among college students in the World Health Organization world mental health surveys. Psychol Med. 2016;46:2955–70. 10.1017/S0033291716001665. 27484622 10.1017/S0033291716001665 PMC5129654

[24] Jacobi F, Höfler M, Strehle J, Mack S, Gerschler A, Scholl L, et al. Psychische störungen in der allgemeinbevölkerung: studie Zur gesundheit erwachsener in Deutschland und Ihr zusatzmodul Psychische gesundheit (DEGS1-MH). Nervenarzt. 2014;85:77–87. 10.1007/s00115-013-3961-y. 24441882 10.1007/s00115-013-3961-y

[25] Zavlin D, Jubbal KT, Noé JG, Gansbacher B. A comparison of medical education in Germany and the united states: from applying to medical school to the beginnings of residency. Ger Med Sci. 2017. 10.3205/000256. 10.3205/000256 PMC5617919 29051721

[26] Negash S, Horn J, Heumann E, Stock C, Zeeb H, Pischke CR, et al. University students’ financial situation during COVID-19 and anxiety and depressive symptoms: results of the COVID-19 German student Well-Being study (C19 GSWS). Psychol Res Behav Manag. 2024;17:2271–85. 10.2147/PRBM.S453694. 38860194 10.2147/PRBM.S453694 PMC11162964

[27] Soh N, Ma C, Lampe L, Hunt G, Malhi G, Walter G. Depression, financial problems and other reasons for suspending medical studies, and requested support services: findings from a qualitative study. Australas Psychiatry. 2012;20:518–23. 10.1177/1039856212460737. 23018118 10.1177/1039856212460737

[28] Ross S, Cleland J, Macleod MJ. Stress, debt and undergraduate medical student performance. Med Educ. 2006;40:584–9. 10.1111/j.1365-2929.2006.02448.x. 16700775 10.1111/j.1365-2929.2006.02448.x

[29] McMichael B, Lee Iv A, Fallon B, Matusko N, Sandhu G. Racial and socioeconomic inequity in the financial stress of medical school. MedEdPublish (2016). 2022;12:3. 10.12688/mep.17544.2. 10.12688/mep.17544.2 PMC9370082 36168540

[30] Armstrong M, Reynolds K. Assessing burnout and associated risk factors in medical students. J Natl Med Assoc. 2020;112:597–601. 10.1016/j.jnma.2020.05.019. 32680700 10.1016/j.jnma.2020.05.019

[31] Deutsches Studentenwerk (DSW). 22. Sozialerhebung. Die wirtschaftliche und soziale Lage der Studierenden. 2023. https://www.studierendenwerke.de/themen/hochschulpolitik/sozialerhebung. Accessed 24 Feb 2025.

[32] Statista. BAföG beziehende Studierende bis 2023| Statista. 10.06.2025. https://de.statista.com/statistik/daten/studie/219/umfrage/anzahl-der-bafoeg-gefoerderten-studenten/. Accessed 10 Jun 2025.

[33] Richardson T, Elliott P, Roberts R, Jansen M. A longitudinal study of financial difficulties and mental health in a National sample of British undergraduate students. Community Ment Health J. 2017;53:344–52. 10.1007/s10597-016-0052-0. 27473685 10.1007/s10597-016-0052-0 PMC5337246

[34] Lange C, Byrd M. The relationship between perceptions of financial distress and feelings of psychological Well-being in new Zealand university students. Int J Adolescence Youth. 1998;7:193–209. 10.1080/02673843.1998.9747824.

[35] Selenko E, Batinic B. Beyond debt. A moderator analysis of the relationship between perceived financial strain and mental health. Soc Sci Med. 2011;73:1725–32. 10.1016/j.socscimed.2011.09.022. 22019305 10.1016/j.socscimed.2011.09.022

[36] Tedstone Doherty D, Kartalova-O’Doherty Y. Gender and self-reported mental health problems: predictors of help seeking from a general practitioner. Br J Health Psychol. 2010;15:213–28. 10.1348/135910709X457423. 19527564 10.1348/135910709X457423 PMC2845878

[37] Vesga-López O, Schneier F, Wang S, Heimberg R, Liu S-M, Hasin DS, Blanco C. Gender differences in generalized anxiety disorder: results from the National epidemiologic survey on alcohol and related conditions (NESARC). J Clin Psychiatry. 2008;69:1606–16. 19192444 PMC4765378

[38] Xu Y, Schneier F, Heimberg RG, Princisvalle K, Liebowitz MR, Wang S, Blanco C. Gender differences in social anxiety disorder: results from the National epidemiologic sample on alcohol and related conditions. J Anxiety Disord. 2012;26:12–9. 10.1016/j.janxdis.2011.08.006. 21903358 10.1016/j.janxdis.2011.08.006

[39] Mackenzie CS, Gekoski WL, Knox VJ. Age, gender, and the underutilization of mental health services: the influence of help-seeking attitudes. Aging Ment Health. 2006;10:574–82. 10.1080/13607860600641200. 17050086 10.1080/13607860600641200

[40] Sagar-Ouriaghli I, Godfrey E, Graham S, Brown JSL. Improving mental health help-seeking behaviours for male students: a framework for developing a complex intervention. Int J Environ Res Public Health. 2020;17: 4965. 10.3390/ijerph17144965. 32660145 10.3390/ijerph17144965 PMC7400593

[41] Tyson P, Wilson K, Crone D, Brailsford R, Laws K. Physical activity and mental health in a student population. J Ment Health. 2010;19:492–9. 10.3109/09638230902968308. 20812852 10.3109/09638230902968308

[42] Grasdalsmoen M, Eriksen HR, Lønning KJ, Sivertsen B. Physical exercise, mental health problems, and suicide attempts in university students. BMC Psychiatry. 2020;20:175. 10.1186/s12888-020-02583-3. 32299418 10.1186/s12888-020-02583-3 PMC7164166

[43] Levine GN, Cohen BE, Commodore-Mensah Y, Fleury J, Huffman JC, Khalid U, et al. Psychological health, Well-Being, and the Mind-Heart-Body connection: A scientific statement from the American heart association. Circulation. 2021;143:e763–83. 10.1161/CIR.0000000000000947. 33486973 10.1161/CIR.0000000000000947

