# Supplementary material for: Mental health and financial concerns in medical students: insights from a cross-sectional study in Germany
Source: BMC Med Educ. 2025 Oct 2;25:1273. doi: 10.1186/s12909-025-07723-5 (PMC12490077; doi:10.1186/s12909-025-07723-5)
Supplement: Supplementary file 1 — Supplementary Material 1. [file 12909_2025_7723_MOESM1_ESM.docx]

**Appendix 1:** Questionnaire finCoHST study (only questions analysed in this article)

- Sociodemographic characteristics:
- How old are you? (freetext)
- Which sex was recorded on your birth certificate? (male/female)
- In which country were you born? In which country was your mother born? In which country was your father born? (in Germany/in another country)
- Have you already completed vocational training or a university degree? (Yes, completed university degree/ Yes, completed vocational training/ Yes, completed university degree AND completed vocational training/ No)
- Study-related aspects:
- In which semester are you in? (1-13)
- Financial concerns (7-point Likert-scale: "strongly disagree" to "strongly agree") according to Jessop et al. [8, 14]:
- I find paying bills economically difficult.
- I have seriously considered abandoning my course because of financial difficulties.
- I worry about my financial situation.
- Financial problems cause me to lose sleep.
- I would list financial difficulties as one of the major stresses in my life at the moment.
- It concerns me that my financial situation means that I miss out on social activities.
- Do you have financial debt? (Yes/ No)
- General physical health:
- Now, I will ask you questions about your physical health: How would you describe your general health status? (Very good, good, fair, poor, very poor)
- Mental health (5-point Likert-scale from "always" to "never") according to the MHI-5:
- How often have you felt happy in the last 4 weeks?
- How often have you felt calm and relaxed in the last 4 weeks?
- How often have you felt nervous in the last 4 weeks?
- How often have you felt discouraged and sad in the last 4 weeks?
- How often have you felt so downhearted that nothing could cheer you up in the last 4 weeks?
